# Suspected Infective Endocarditis in a Premature Neonate After Patent Ductus Arteriosus Occluder Placement: A Case Report

**DOI:** 10.1155/crpe/6636016

**Published:** 2026-10-07

**Authors:** Kiely Fagundes, Megan Murphy, Apthi Muralidhar, Debra Palazzi, Muralidhar Premkumar, Daniela Dinu

**Affiliations:** ^1^ Department of Pediatrics Baylor College of Medicine Houston Texas USA bcm.edu; ^2^ University of Texas Austin Texas USA uta.edu; ^3^ Department of Pediatrics Section of Infectious Diseases Baylor College of Medicine Houston Texas USA bcm.edu; ^4^ Department of Pediatrics Section of Neonatology Baylor College of Medicine Houston Texas USA bcm.edu

**Keywords:** bacteremia, case report, PDA occluder, *Staphylococcus capitis*

## Abstract

Patent ductus arteriosus (PDA) is the most common cardiac abnormality in premature neonates, and percutaneous, transcatheter occlusion devices are increasingly used for its nonsurgical closure. Even though PDA occluders are safe and effective, they are not without risks. Infective endocarditis (IE), a complication associated with percutaneous occlusion device placement in other congenital heart conditions, has not been described with a PDA occluder. We describe a case of suspected endocarditis in an extremely low birth weight (ELBW) neonate after PDA occluder placement.

## 1. Background

Patent ductus arteriosus (PDA) is the most common cardiac abnormality in neonates, and its incidence is inversely correlated with the gestational age (GA), with 80% of neonates born before 28‐week GA and 90% of those born before 24‐week GA having a PDA at 4 days of life [1]. Significant morbidities are associated with hemodynamically significant PDA, namely, bronchopulmonary dysplasia, necrotizing enterocolitis, intraventricular hemorrhage, renal dysfunction, pulmonary hemorrhage, and death [1, 2]. Different treatment options for PDA include medical closure using nonsteroidal anti‐inflammatory drugs (indomethacin, ibuprofen), and, more recently, acetaminophen, mechanical occlusion using transcatheter devices, and transthoracic surgical closure. Multiple devices have been used since the first description of transcatheter device‐based PDA occlusion with the latest to receive Food and Drug Administration (FDA) approval being the Amplatzer Piccolo . It is used in premature infants with birth weight as low as 700 g [3] and has been demonstrated to be safe and effective [3, 4] with hypothesized potential avoidance of adverse outcomes associated with prolonged PDA patency. Additional devices have been used off‐label for transcatheter closure in premature infants such as the Medtronic Microvascular Plug (MVP; Medtronic, MN) and the KA Medical Micro Plug (KA Medical, MN) [5]. However, percutaneous device closure is associated with risks such as device malposition, residual shunt, left pulmonary arterial stenosis, lower limb ischemia when arterial access is needed, embolization, infective endocarditis (IE), and the narrowing of adjacent vessels [4, 6, 7].

IE is estimated to occur annually in 3.3/100,000 infants younger than one year of age [8, 9]. Several predisposing factors have been described, such as congenital heart disease, including PDA, central venous catheters, and prematurity [8, 9]. *Staphylococcus* and *Candida* species are the most commonly implicated pathogens in IE. While there is a lack of data about the precise incidence of IE after PDA occlusive device placement in newborns, this complication has been described in infants and children with other types of congenital heart disease such as atrial septal defects [10, 11].

## 2. Case Presentation

A 25‐week 0‐day dichorionic‐diamniotic female twin was born to a 22‐year‐old mother via repeat Cesarean section following concerns for possible placental abruption in the setting of multiple pregnancy. Apgar scores were 7 at 1 min and 8 at 5 min of life, and resuscitation was routine. Birth weight was 785 g (74.4^th^ percentile), length was 34 cm (88.4^th^ percentile), and head circumference was 22 cm (41.5th percentile). Physical examination was normal for GA. On postnatal day (PND) 1 (with day of birth defined as PND 0), she was intubated for surfactant administration and received vasopressors for hypotension. On PND 2, fresh bloody secretions were noted in the endotracheal tube, consistent with pulmonary hemorrhage. On PND 4, hypotension worsened and echocardiogram demonstrated a large PDA with continuous left‐to‐right shunting. Due to prolonged need for parenteral nutrition and vasopressor medication, a peripherally inserted central catheter (PICC) was placed on PND 5. Following medical treatment with ibuprofen standard dose of 10 mg, 5 mg, and 5 mg, respectively, administered intravenously from PND 5 through 7 for PDA closure, a subsequent echocardiogram obtained on PND 9 redemonstrated a large PDA, unchanged from the previous one.

Despite medical management for PDA closure, her lung disease and hypotension worsened resulting in increasing requirement in mechanical ventilation, vasopressor medications, and blood products. Blood cultures drawn on PND 10 were negative, and the infant received empiric antibiotics for 48 h. Repeat echocardiogram on PND 17 demonstrated an unchanged, large PDA with continuous low velocity left to right shunting. Conservative management of the PDA with fluid restriction (140 mL/kg/day) was continued, and furosemide was added. On PND 18, blood cultures were drawn due to worsening respiratory status and grew coagulase‐negative staphylococci (CoNS). The infant was treated with a 7‐day course of vancomycin, achieving clearance of bacteremia. Due to the ongoing need for central venous access to provide adequate nutrition in this critically ill extremely low birth weight (ELBW) infant, the infection was treated without removal of the PICC. Though the infant showed some improvement in respiratory status, an echocardiogram on PND 24 showed a persistent hemodynamically significant PDA. Due to the persistent need for ventilatory support and poor growth, the Pediatric Cardiology service was consulted. Based on the small weight and tenuous respiratory status, a catheter‐based procedure was recommended, as opposed to a surgical ligation of the PDA which would have involved thoracotomy with associated risks. A repeat course of medical treatment for PDA closure was not considered due to lower likelihood of successful closure at this chronological age. Cardiac catheterization and transcutaneous PDA occlusion was completed on PND 31 with a KA 5‐mm device. At the time of catheterization, her weight was 1.12 kg (45.5^th^ percentile) and length was 36.7 cm (32.8^th^ percentile). Access to the PDA was obtained through the right heart, pulmonary artery, across the PDA and into aorta. The device was deployed, and a transthoracic echocardiogram showed well‐seated device with no residual ductal flow and no obstruction of the aorta and left pulmonary artery. An additional echocardiogram performed one day after procedure had similar findings and no migration of the device. Subsequently, the infant was successfully extubated on PND 47 following treatment with steroids.

On PND 53, a sepsis evaluation performed following another clinical deterioration revealed gram‐positive cocci in clusters, later identified as *Staphylococcus capitis*. Initial antibiotics included nafcillin and amikacin, with introduction of vancomycin following the identification of the organism. A left lower extremity Doppler ultrasound showed a PICC‐associated occlusive thrombus in the left greater saphenous vein. Though the PICC was removed on PND 54, blood cultures performed on PND 56 were positive for *Staphylococcus capitis* after two previous negative blood cultures on PND 54 and 55 (Figure 1). The susceptibility results of both blood cultures were similar, indicating sensitivity to vancomycin and rifampin. An echocardiogram showed no evidence of vegetation or intracardiac lesions. No further imaging studies were performed, and the tip of the PICC line was not cultured, as this is not done at our institution. Based on recommendations from the Infectious Diseases Service, a 42‐day course of antibiotics (vancomycin and rifampin) with 14 days of amikacin for a synergistic effect was completed on PND 99. There were two additional negative blood cultures on PND 56 and 57 to document resolution of the infection. The patient was discharged home on PND 124. No further evaluations for bacterial infection were required during the admission.

**FIGURE 1:**
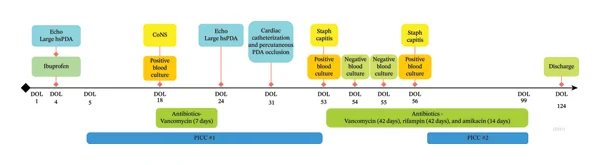
Clinical course with pertinent events.

Outpatient follow‐up visits at 5 months of age (2 months after discharge) and 16 months of age demonstrated a normal electrocardiogram and a well‐seated PDA occluder without residual PDA shunt on echocardiography. Currently, the infant has no cardiac restrictions, does not require SBE prophylaxis, and is followed by cardiology.

## 3. Discussion

The management of the PDA in premature infants remains controversial, with a lack of consensus on the necessity, timing (prophylactic vs. therapeutic), and method of closure (medical, catheter‐based, or surgical). The advent of a new device (Piccolo occluder), which can be safely placed in very small and immature preterm infants using a minimally invasive technique, has been associated with a high success rate and low procedural morbidity profile [3, 4]. Similar devices, commonly used off‐label in premature infants, have shown exceptional success and low procedural morbidity [5], including the device used in our case. Larger devices have been used for decades in both adults and children, for catheter‐based atrial septal defect and PDA closure. Catheter‐based procedures are preferred by both parents [12] and providers as suggested by the increased use [13], and they have been recommended as the favored approach over PDA ligation in institutions where sufficient capability from the perspective of personnel, experience, and center volume is available, and patient characteristics are appropriate [14]. However, they do have short‐ and long‐term complications such as device malposition, embolization, arrhythmias, thrombosis, IE, and hemolysis [4, 5].

IE as a complication of ASD and PDA occlusive devices is rare, in both adults and children [10, 15]. The literature suggests that device‐related IE may occur through either bacteremia from the puncture site at the time of insertion or seeding of bacteria before or after device insertion [11]. In both adults and pediatric patients, *Staphylococcus aureus* remains the most common bacterial etiology [11]. CoNS, including *Staphylococcus capitis*, are less commonly reported as etiologic agents of IE. Such infections are mostly described in patients with prosthetic valves, pacemakers [16, 17], and rarely on native valves [18]. However, CoNS, including *Staphylococcus capitis*, are common pathogenic agents causing late‐onset bacteremia in neonatal intensive care units (NICUs) particularly in preterm babies [19]. These pathogens have the ability to form biofilms on the surface of inserted devices.

*Staphylococcus capitis* is part of the normal flora of the skin of the scalp, face, ears, and neck and accounts for 5% of pathogenic isolates of CoNS. It has been shown to cause pneumonia, urinary tract infection, catheter‐related bacteremia, and cellulitis [18]. It is likely that the number of *S. capitis*‐related infections is underestimated given the fact that CoNS is not systematically speciated and is frequently clustered as CoNS in clinical studies.

In our case, several risk factors for IE were present, including prematurity, ELBW, and the presence of foreign devices such as a PICC line and a PDA occluder device [8, 9]. Persistent *Staphylococcus capitis* bacteremia 3 days after PICC removal raised concern for an alternative source of infection unrelated to central venous access. The PDA occluder, being synthetic, could serve as a potential nidus for infection. An infected thrombus with possible embolization was also considered. Irrespective of these considerations, in this context, most clinicians would adopt a cautious approach and manage the situation as suspected device infection or endocarditis with prolonged antibiotics.

We recommend considering catheter tip cultures and additional imaging in similar cases. Infections caused by organisms typically regarded as contaminants should not be dismissed without careful evaluation. Greater scrutiny of such cases may help determine whether device seeding is occurring. Currently, there are no guidelines for treating premature infants with proven or suspected IE with CoNS in the presence of prosthetic material. Treatment recommendations are derived from the Infectious Diseases Society of America provided for adult patients. In adults with IE secondary to CoNS in the presence of prosthetic material, the guidelines recommend dual therapy with vancomycin and rifampin for a minimum duration of 6 weeks, along with amikacin added for synergistic effect during the first 2 weeks. It is also recommended that the patient be monitored carefully for the development of periannular extension or extracardiac spread of infection.

## 4. Conclusion

Premature infants are at high risk for the development of late‐onset sepsis. While catheter‐related blood stream infections are common, other rare causes of persistent bacteremia should be considered. In infants with various prosthetic devices, extended antibiotic therapy may be prudent. CoNS, including *Staphylococcus capitis*, have been increasingly described as etiologic agents of endocarditis in older patients, on both native and prosthetic valves and implantable devices, and should be considered in NICU patients.

## Funding

The authors have nothing to report.

## Ethics Statement

Institutional Review Board (IRB) approval was not required for this single anonymized case report. The requirement for written informed consent for publication was waived by the institutional ethics committee because all patient information was de‐identified, and no identifiable or potentially identifiable information is included in this manuscript.

## Conflicts of Interest

The authors declare no conflicts of interest.

## Supporting Information

Additional supporting information can be found online in the Supporting Information section.

## Supporting information


**Supporting Information** CARE guidelines v2.

## Data Availability

The data that support the findings of this study are available on request from the corresponding author. The data are not publicly available due to privacy or ethical restrictions.
