# Supplementary material for: Suspected Infective Endocarditis in a Premature Neonate After Patent Ductus Arteriosus Occluder Placement: A Case Report
Source: Case Rep Pediatr. 2026 Oct 7;2026:6636016. doi: 10.1155/crpe/6636016 (PMC13646714; doi:10.1155/crpe/6636016)
Supplement: Supplementary file 1 [file CRPE-2026-6636016-s001.pdf]

CARE case report guidelines

CARE Checklist of information to include when writing a case report

(cc) BY-NC-ND

Item	Checklist item description	Reported on Line
1	The diagnosis or intervention of primary focus followed by the words “case report”	1
2	2 to 5 key words that identify diagnoses or interventions in this case report, including "case report"	1
3a	Introduction: What is unique about this case and what does it add to the scientific literature?	3
3b	Main symptoms and/or important clinical findings	4
3c	The main diagnoses, therapeutic interventions, and outcomes	4
3d	Conclusion—What is the main “take-away” lesson(s) from this case?	5
4	One or two paragraphs summarizing why this case is unique (**may include references**)	5
5a	De-identified patient specific information	4
5b	Primary concerns and symptoms of the patient	4
5c	Medical, family, and psycho-social history including relevant genetic information	4
5d	Relevant past interventions with outcomes	N/A
6	Describe significant physical examination (PE) and important clinical findings	4
7	Historical and current information from this episode of care organized as a timeline	8
8a	Diagnostic testing (such as PE, laboratory testing, imaging, surveys)	4
8b	Diagnostic challenges (such as access to testing, financial, or cultural)	N/A
8c	Diagnosis (including other diagnoses considered)	4
8d	Prognosis (such as staging in oncology) where applicable	N/A
9a	Types of therapeutic intervention (such as pharmacologic, surgical, preventive, self-care)	4
9b	Administration of therapeutic intervention (such as dosage, strength, duration)	4
9c	Changes in therapeutic intervention (with rationale	N/A
10a	Clinician and patient-assessed outcomes (if available)	5
10b	Important follow-up diagnostic and other test results	5
10c	Intervention adherence and tolerability (How was this assessed?)	4
10d	Adverse and unanticipated events	4-5
11a	A scientific discussion of the strengths AND limitations associated with this case report	5-6
11b	Discussion of the relevant medical literature **with references**	5-6
11c	The scientific rationale for any conclusions (including assessment of possible causes)	5-6
11d	The primary “take-away” lessons of this case report (without references) in a one paragraph conclusion	6
12	The patient should share their perspective in one to two paragraphs on the treatment(s) they received	No
13	Did the patient give informed consent? Please provide if requested	Yes No x
